# Transcriptome Analysis Provides New Insight into Apoptosis and Immunosuppression in *Procambarus clarkii* After Exposure to High Temperature

**DOI:** 10.3390/biology15070582

**Published:** 2026-04-05

**Authors:** Shengjie Ren, Wenjing Xu, Xianjun Ma, Qin Gui, Wanqiu Tian, Qiuning Liu, Lishang Dai, Dandan Bian

**Affiliations:** 1 State Key Laboratory Incubation Base for Conservation and Utilization of Bio-Resource in Tarim Basin, Tarim University, Alaer 843300, China; renshengjie1990@163.com (S.R.); 13139803605@163.com (W.X.); 19590120700@163.com (Q.G.); 19942287674@163.com (W.T.); 2 College of Life Science and Agriculture Forestry, Qiqihar University, Qiqihar 161006, China; 3 School of Wetlands, Yancheng Teachers University, Yancheng 224007, China; liuqn@yctu.edu.cn; 4 School of Pharmaceutical Sciences, Wenzhou Medical University, Wenzhou 325027, China; dls1987@126.com

**Keywords:** *Procambarus clarkii*, heat stress, oxidative stress, endoplasmic reticulum stress, hemocyte apoptosis, immunosuppression, transcriptomics

## Abstract

Acute heat stress frequently causes mass mortality in farmed red swamp crayfish (*Procambarus clarkii*), yet the mechanisms underlying immune collapse remain poorly understood. We established an acute heat stress model (37 °C, 6 h) and performed an integrative analysis combining hemocyte profiling, redox and immune assays, RNA-seq, and qRT-PCR. Heat stress significantly increased mortality and disrupted the hemocyte system, with a ~25% reduction in total hemocyte count and a selective decline in granular cells. This was associated with severe redox imbalance, evidenced by ROS/H_2_O_2_ accumulation, suppressed SOD and CAT activities, and lipid peroxidation damage. Transcriptomic analysis revealed 1446 differentially expressed genes, indicating concurrent activation of ER stress and autophagy alongside suppression of energy metabolism. Key gene validation confirmed upregulation of pro-apoptotic factors (*CASP3*, *P53*) and ER stress markers (*GRP78*, *XBP1*), consistent with hemocyte depletion. These findings provide multi-level evidence that acute heat stress triggers a redox crisis (“oxidative burst–defense suppression”), which in turn activates ER stress and apoptosis, leading to selective loss of granular cells and systemic immune compromise. This study establishes a mechanistic framework for understanding heat-induced mortality in crustaceans and offers a theoretical basis for developing targeted interventions to enhance thermal resilience in crayfish aquaculture.

## Simple Summary

This study investigates how acute heat stress causes immune failure in farmed red swamp crayfish. Using a 37 °C heat stress model, we found that heat stress increased mortality, disrupted hemocyte balance, and induced severe oxidative stress. Transcriptomic analysis revealed activation of ER stress and autophagy alongside metabolic suppression. Key gene changes confirmed that heat stress triggers oxidative damage and cell death, leading to immune collapse. These findings provide important insights into heat-induced mortality in crustaceans.

## 1. Introduction

The red swamp crayfish (*Procambarus clarkii*) is a cornerstone species in global freshwater aquaculture and a key contributor to food security [1,2]. As a poikilotherm, its physiology and survival are highly sensitive to environmental temperature [3]. Consequently, climate change-driven increases in the frequency and intensity of extreme heat events pose a major threat to its sustainable cultivation [4,5]. In key production regions such as China’s Yangtze River basin, water temperatures exceeding 35 °C have been associated with 20–30% mortality spikes [6], resulting in substantial economic losses. Although the lethal effects of high temperature are well documented, the precise cellular and molecular mechanisms underlying heat-induced mortality remain incompletely understood, impeding the development of effective mitigation strategies.

The crustacean innate immune system, comprising cellular (hemocytes) and humoral components [7,8], serves as the primary defense against pathogens, yet is highly vulnerable to thermal stress [4,9]. Across species, heat stress broadly compromises immune competence [7,8,9,10], leading to reduced hemocyte counts [2,11], diminished phenoloxidase activity [12,13], and increased susceptibility to pathogens [14,15,16]. This heat-induced immunosuppression is widely considered a key contributor to thermal mortality, supported by studies in related crustaceans where heat stress is associated with hemocyte apoptosis and suppression of antioxidant defenses [17,18].

In *P. clarkii*, however, the molecular drivers of this systemic immune collapse remain poorly defined. While phenotypic studies have documented metabolic disturbances [19] and enhanced vulnerability to pollutants under thermal stress [20,21,22], the underlying signaling pathways and decisive cellular events leading to immune failure have yet to be characterized. This represents a critical knowledge gap: how elevated temperature subverts immune function at the molecular level remains unclear.

Emerging evidence supports a model of “multi-pathway synergistic” impairment, in which heat stress orchestrates immune collapse through coordinated molecular networks [3,13,23]. Proposed mechanisms include the induction of heat shock proteins that may suppress NF-κB-mediated immune responses [24], and reactive oxygen species (ROS) bursts that activate pivotal pathways such as p53 [25] and MAPK [26], concurrently regulating apoptosis and immune function [24,27]. Transcriptomic analyses further reveal broad heat-induced dysregulation of immune, apoptotic [19], and metabolic genes [28], including downregulation of specific antimicrobial peptides [3,13,24]. Despite these insights, an integrative understanding linking the initial thermal signal to the final immune breakdown remains lacking.

To address this critical gap, we hypothesized that acute heat stress induces systemic immune compromise in *P. clarkii* through an oxidative damage-centered mechanism in hemocytes, with this oxidative insult serving as a central initiating event. To test this hypothesis, we employed an integrated approach combining controlled heat stress with hemocyte profiling, oxidative and ER stress assessment, and transcriptomic analysis. Specifically, we aimed to explore the mechanisms by which heat may suppress immune effector molecules via key signaling hubs, assess whether apoptosis is a principal correlate of heat-induced hemocyte loss, and evaluate the relative contributions of mitochondrial dysfunction versus ER stress within this cascade. Collectively, this study delineates an “immune–oxidative–cell death” network and defines its key regulatory nodes in *P. clarkii*, providing a mechanistic framework that may guide the development of targeted interventions to enhance thermal tolerance in crustacean aquaculture under climate change.

## 2. Materials and Methods

### 2.1. Experimental Animals and Heat Stress Procedure

Sexually mature red swamp crayfish (*P. clarkii*) in the intermolt stage were obtained from a commercial farm in Xuyi County, China. Animals were acclimated for two weeks in a recirculating aquaculture system under standard conditions: temperature (25.0 ± 0.5) °C, pH 7.4 ± 0.2, dissolved oxygen > 5.0 mg/L, ammonia-N < 0.01 mg/L, and a 12 h light:12 h dark photoperiod. Following acclimation, crayfish with a body weight of 9.55 ± 1.24 g were randomly distributed into six 50 L tanks (40 crayfish per tank).

**Figure 1:**
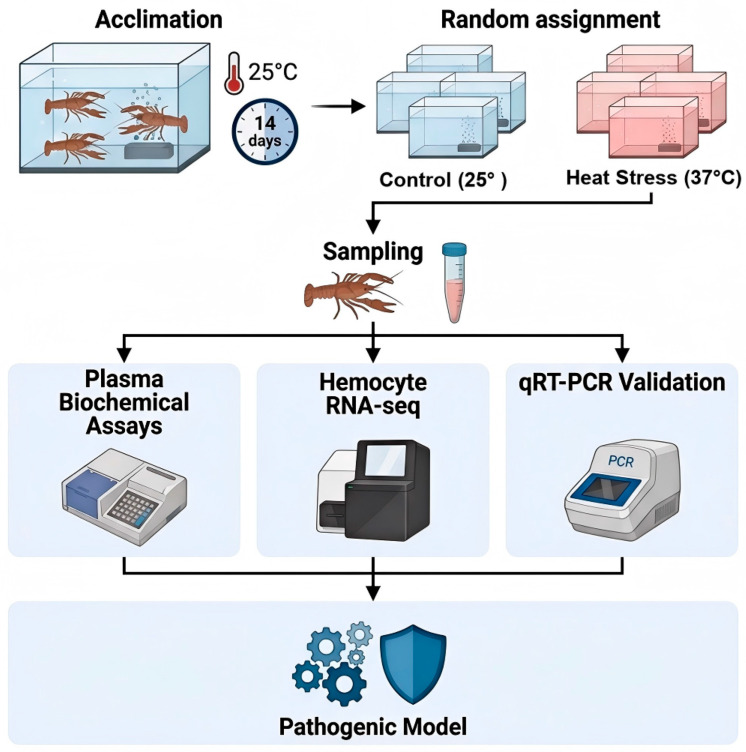
Schematic diagram of the experimental design. Crayfish (*P. clarkii*) were acclimated for 14 days at 25 °C, followed by random assignment to a control group (maintained at 25 °C) and a heat stress group (exposed to 37 °C for 6 h). Following the exposure period, hemolymph was collected for plasma and hemocyte isolation. Hemocytes were subjected to RNA-seq and qRT-PCR validation, while plasma was used for biochemical assays. These datasets were then integrated to construct a pathogenic model elucidating the mechanisms of heat-induced immune collapse.

The tanks were assigned to two groups (three replicate tanks per group): a control group maintained at (25.0 ± 0.5) °C, and a heat stress (HS) group. For the HS group, water temperature was increased from 25.0 °C to 37.0 °C at a rate of 1.0 °C/h using a programmable thermostat (Model TH21, LABSTAR, Karlsruhe, Germany; accuracy ±0.1 °C) and then maintained at 37.0 °C for 6 h. This target temperature was selected to induce acute thermal stress based on prior studies [27,29]. Water temperature was monitored every 2 h with a calibrated thermometer. All other water quality parameters were maintained as during acclimation. No significant tank-to-tank variation was observed in preliminary analyses. The overall experimental design is summarized in Figure 1.

### 2.2. Sample Collection

Following the 6 h exposure period, samples were collected concurrently from the control and HS groups. Following the 6 h exposure period, samples were collected concurrently from the control and HS groups. Among these, hemolymph from nine individuals (three per tank) was processed for plasma and hemocyte isolation, while hemolymph from the remaining nine individuals (three per tank) was reserved exclusively for total hemocyte count (THC).

All selected crayfish were anesthetized on ice for 15 min. Approximately 2 mL of hemolymph was aspirated from the pericardial cavity using a sterile syringe pre-rinsed with a modified anticoagulant solution (per 100 mL: 0.2 g NaCl, 0.8 g citric acid, 7.65 g trisodium citrate, 2.05 g glucose, pH adjusted to 4.6, filter-sterilized). The hemolymph was immediately mixed 1:1 (*v*/*v*) with the same ice-cold anticoagulant in a pre-chilled tube.

### 2.3. Hemolymph Processing and Hemocyte Counts

For the nine individuals designated for biochemical and molecular analyses, the hemolymph–anticoagulant mixture was centrifuged at 4 °C, 5000× *g* for 10 min. The supernatant (plasma) was aliquoted and stored at −80 °C [30,31]. The hemocyte pellet was gently washed with ice-cold sterile PBS (0.01 M, pH 7.4), centrifuged again under identical conditions, and the final pellet was flash-frozen in liquid nitrogen for subsequent transcriptomic analysis. To minimize degradation of oxidative markers, all plasma samples were assayed within one month of collection.

For the nine individuals designated for THC, the diluted hemolymph was loaded onto a hemocytometer and counted immediately after collection [32,33]. A 10 μL aliquot of the diluted hemolymph (1:1 with anticoagulant) was loaded onto an improved Neubauer hemocytometer (BRAND, Wertheim, Germany). After 1–2 min of settlement, hemocytes were counted under an optical microscope (Olympus CX23, Tokyo, Japan) at 400× magnification across the four large corner squares. THC was calculated as: THC (cells/mL) = (N/4) × 2 × 10^4^, where N is the total number of hemocytes counted in the four squares, and 2 is the dilution factor.

For differential hemocyte analysis, a fixed volume of the hemocyte pellet was resuspended and fixed with 4% paraformaldehyde in PBS for 2 h at room temperature. Smears were prepared on glass slides, air-dried, fixed in methanol for 3 min, and stained with Wright-Giemsa for 10 min. After rinsing with phosphate buffer (pH 6.8), slides were air-dried. Stained smears were examined under oil immersion (1000×) using a light microscope. Hemocytes were classified into hyalinocytes, semigranulocytes, and granulocytes based on established morphological criteria [34,35]. For each biological replicate, at least 200 cells were counted across five randomly selected, non-overlapping fields of view. Results were expressed as the percentage of each hemocyte type [36,37].

### 2.4. Biochemical Assays

All biochemical assays were conducted in duplicate using an Infinite M1000 Pro microplate reader (Tecan, Männedorf, Switzerland). Commercial kits (Nanjing Jiancheng Bioengineering Institute, Nanjing, China) were used to measure antioxidant enzymes, immune-related enzymes, and oxidative metabolites according to the manufacturer’s protocols. Antioxidant enzyme activities, including total superoxide dismutase (T-SOD, Cat# A001-3-2), catalase (CAT, Cat# A007-1-1), and glutathione peroxidase (GSH-Px, Cat# A005-1-2), were determined. Immune-related enzyme activities, namely acid phosphatase (ACP, Cat# A060-2-2), alkaline phosphatase (AKP, Cat# A059-2-2), and lysozyme (LZM, Cat# A005-1-1), were also assessed. Oxidative metabolites, including hydrogen peroxide (H_2_O_2_, Cat# A064-1-1) and malondialdehyde (MDA, Cat# A003-1-2), were quantified.

Absorbance readings were converted to concentrations or activities using assay-specific standard curves. All final data were normalized to the total protein content of the plasma, as determined by a bicinchoninic acid (BCA) protein assay kit. Additionally, the relative level of reactive oxygen species (ROS) was quantified using a sandwich ELISA kit (Cat# YS02255B, Yaji Biotechnology, Shanghai, China). Briefly, diluted plasma samples were incubated in pre-coated wells at 37 °C, followed by sequential addition of enzyme-conjugated antibodies and TMB substrate. The reaction was stopped, and the absorbance at 450 nm was measured.

### 2.5. Transcriptomic Sequencing and Analysis

#### 2.5.1. RNA Extraction, Quality Control, and Library Preparation

Total RNA was extracted from snap-frozen hemocyte pellets using TRIzol reagent (Invitrogen, Waltham, MA, USA). RNA purity was assessed with a NanoDrop 2000 spectrophotometer (Thermo Fisher Scientific, Waltham, MA, USA); samples with A260/A280 between 1.8 and 2.2 and A260/A230 ≥ 2.0 were accepted. RNA integrity was evaluated using an Agilent 2100 Bioanalyzer with the RNA Nano 6000 kit (Agilent Technologies, Santa Clara, CA, USA). Only samples with concentration ≥ 35 ng/μL, total amount ≥ 1 μg, and RNA Integrity Number (RIN) ≥ 7.0 were used for library construction.

Strand-specific cDNA libraries were constructed using the NEBNext^®^ Ultra™ II Directional RNA Library Prep Kit for Illumina (NEB, Ipswich, MA, USA). Briefly, mRNA was enriched with Oligo(dT) beads, fragmented, and used for first- and second-strand cDNA synthesis. After end repair, adenylation, and adapter ligation, fragments of approximately 350 bp were selected with AMPure XP beads (Beckman Coulter, Indianapolis, IN, USA) and enriched by PCR. Libraries were quantified with a Qubit 4.0 fluorometer and validated on the Agilent 2100 Bioanalyzer (Santa Clara, CA, USA). Sequencing was performed on an Illumina NovaSeq 6000 platform (Beijing, China), generating 150 bp paired-end reads.

#### 2.5.2. Bioinformatics Analysis

Raw reads were processed with fastp (v0.23.2) [38] to remove adapters and low-quality bases (Qphred ≤ 20). Clean reads were aligned to the *P. clarkii* reference genome (NCBI accession: GCF_020424385.1) using HISAT2 (v2.2.1) [39]. Gene expression was quantified as FPKM using featureCounts (v2.0.3) (v2.0.3) [40].

Differential expression analysis between the control and HS groups was performed with the DESeq2 R package (v1.38.3) [38,41]. Genes with |log_2_ fold change| ≥ 1 and false discovery rate (FDR) < 0.05 were considered significantly differentially expressed (DEGs). Principal component analysis (PCA) was conducted on variance-stabilized transformed counts.

For functional interpretation, DEGs were subjected to Gene Ontology (GO) and Kyoto Encyclopedia of Genes and Genomes (KEGG) pathway enrichment analyses using clusterProfiler (v4.6.2) [42]. Terms and pathways with FDR < 0.05 were considered significant.

### 2.6. Quantitative Real-Time PCR Analysis 

Total RNA was extracted from hemocytes (three biological replicates per group) using a commercial kit (Vazyme Biotech, Nanjing, China). RNA quality was verified by spectrophotometry (A260/A280: 1.9–2.1) and gel electrophoresis (intact 28S/18S rRNA bands). One microgram of RNA was reverse-transcribed into cDNA using the PrimeScript RT Kit with gDNA Eraser (TaKaRa Bio, Dalian, China).

qRT-PCR was performed in technical triplicate on a LightCycler 480 system (Roche, Indianapolis, IN, USA). Each 10 µL reaction contained 1 µL cDNA, 5 µL 2× ChamQ SYBR Master Mix (Vazyme, Nanjing, China), 0.5 µL each of forward and reverse primers (10 µM; sequences listed in Table 1), and 3 µL nuclease-free water. Cycling conditions were: 95 °C for 30 s; 40 cycles of 95 °C for 5 s and 60 °C for 30 s; followed by melting curve analysis. The 18S rRNA gene was used as the endogenous reference due to its stable expression (coefficient of variation in Ct values < 5% across groups) [37,43,44]. Relative expression levels were calculated using the 2^−ΔΔCt^ method [45].

### 2.7. Statistical Analysis

All statistical analyses were performed using IBM SPSS Statistics 24.0 (IBM Corp., Armonk, NY, USA). Data are presented as mean ± standard deviation (SD) of three independent biological replicates. Differences between the two groups were analyzed by an unpaired two-tailed Student’s *t*-test. Normality of data distribution was assessed, and homogeneity of variances was verified using Levene’s test. When the assumption of equal variances was violated (*p* < 0.05 in Levene’s test), Welch’s corrected *t*-test was used instead. Graphs were generated using GraphPad Prism 8.0 (GraphPad Software, San Diego, CA, USA). Statistical significance was set at α = 0.05 and is denoted in figures as follows: *p* < 0.05, *p* < 0.01, *p* < 0.001; “ns” indicates non-significance (*p* ≥ 0.05).

## 3. Results

### 3.1. Effects of Acute Heat Stress on Hemocyte Parameters and Immune Responses in P. clarkii

**Figure 2:**
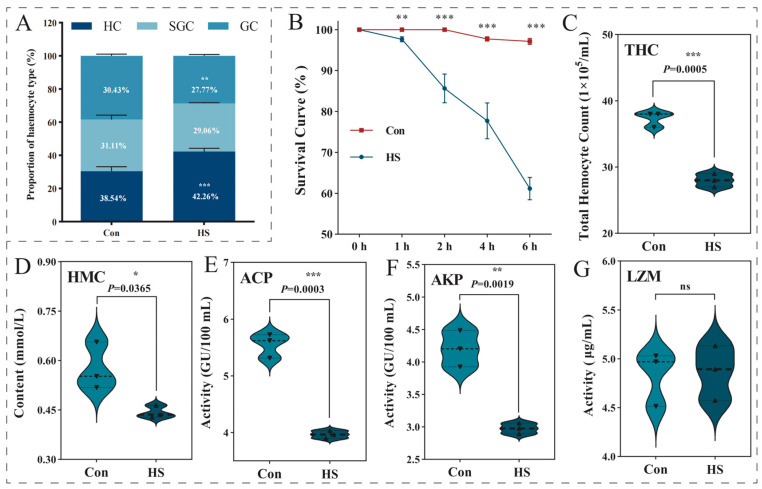
Effects of acute heat stress on survival, hemocyte parameters, and immune enzyme activities in *P. clarkii*. Crayfish were exposed to 37 °C (HS group) for 6 h compared with the control group (Con) maintained at 25 °C. Changes were assessed in: (**A**) hemocyte subpopulation proportions (HC: hyaline cells, SGC: semigranular cells, GC: granular cells, %); (**B**) cumulative mortality; (**C**) total hemocyte count (×10^5^ cells/mL); (**D**) hemocyanin content; (**E**) acid phosphatase (ACP) activity; (**F**) alkaline phosphatase (AKP) activity; and (**G**) lysozyme (LZM) activity. Data are presented as mean ± SD (n = 3). Significance was determined by independent samples *t*-test (*, *p* < 0.05; **, *p* < 0.01; ***, *p* < 0.001; ns, not significant).

A 6 h exposure to 37 °C significantly compromised the immune capacity of *P. clarkii*, as evidenced by alterations in both cellular and humoral immune parameters. Survival analysis using the Kaplan–Meier method revealed that the heat-stressed group experienced significantly higher cumulative mortality compared to the control group throughout the exposure period (log-rank test, *p* < 0.001; Figure 2B)

Acute heat stress was associated with a significant reorganization of the circulating hemocyte population (Figure 2A). The proportion of hyaline cells (HC) increased from 38.54% to 42.26% (*p* < 0.001), while the percentages of semi-granular cells (SGC) and granular cells (GC) decreased from 31.11% to 29.06% and from 30.43% to 27.77%, respectively. Consistent with these shifts, the total hemocyte count (THC) was significantly reduced by approximately 25% in the heat-stressed group (*p* < 0.001; Figure 2C).

In addition to these cellular changes, several humoral immune parameters were significantly altered. Hemolymph hemocyanin (HMC) concentration was significantly lower following heat exposure (*p* < 0.05; Figure 2D). Similarly, the activities of two key lysosomal enzymes, acid phosphatase (ACP) and alkaline phosphatase (AKP), were markedly suppressed (*p* < 0.001 and *p* < 0.01, respectively; Figure 2E,F). Although lysozyme (LZM) activity showed a declining trend, the difference between groups did not reach statistical significance (Figure 2G).

### 3.2. Effects of Acute Heat Stress on Hemolymph Redox Homeostasis in P. clarkii

**Figure 3:**
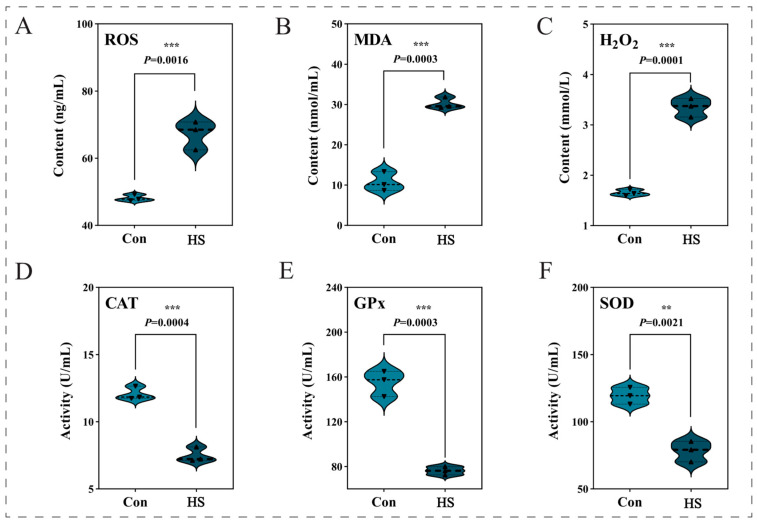
Effects of acute heat stress on hemolymph oxidative stress indicators in *P. clarkii*. Crayfish were exposed to 37 °C or control (Con) conditions for 6 h. Measured parameters included oxidative products (**A**–**C**): reactive oxygen species (ROS), malondialdehyde (MDA), and hydrogen peroxide (H_2_O_2_); and antioxidant enzyme activities (**D**–**F**): catalase (CAT), glutathione peroxidase (GPx), and superoxide dismutase (SOD). Data are mean ± SD (n = 3). Significance was assessed by independent samples t-test (**, *p* < 0.01; ***, *p* < 0.001); exact *p*-values are shown.

Acute heat stress severely disrupted systemic redox homeostasis in *P. clarkii*, leading to oxidative damage and compromised antioxidant defense. Marked accumulation of oxidative products was observed. Hemolymph ROS levels were significantly elevated in the heat-stressed group compared to the control (*p* < 0.001; Figure 3A). Concomitantly, the content of hydrogen peroxide (H_2_O_2_) increased sharply by approximately 1.03-fold (*p* < 0.001; Figure 3C). The level of malondialdehyde (MDA), a terminal lipid peroxidation product, also increased significantly (*p* < 0.001; Figure 3B).

Conversely, the antioxidant enzyme system was broadly inhibited. The activities of catalase (CAT) and glutathione peroxidase (GPx) were reduced by approximately 39% and 51%, respectively (both *p* < 0.001; Figure 3D,E). Furthermore, the activity of superoxide dismutase (SOD) was also significantly lower (*p* < 0.001; Figure 3F).

### 3.3. RNA-Seq of Hemocytes Unveils the Molecular Response to Acute Heat Stress in P. clarkii

To elucidate the molecular mechanisms underlying the response to acute heat stress, RNA-Seq was performed on hemocytes from control (Con) and heat-stressed (HS, 37 °C) groups. A total of approximately 39.09 Gb of clean data were obtained, with Q30 scores exceeding 94.28% across all samples (Tables S1 and S2), supporting the reliability of subsequent analyses. Principal component analysis (PCA) revealed a distinct separation between Con and HS samples along PC1, which explained 76.4% of the variance, with high intra-group reproducibility (Figure S1), indicating a systemic transcriptomic reprogramming induced by heat stress.

**Figure 4:**
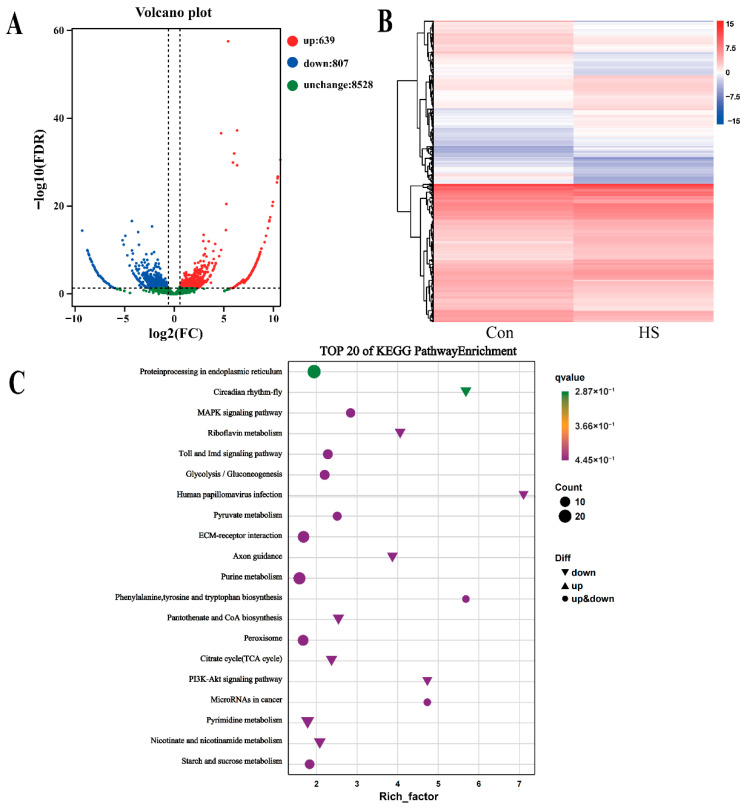
Differential expression analysis and KEGG pathway enrichment of hemocytes under acute heat stress. Transcriptomic profiling of *P. clarkii* hemocytes from control (Con) and 6 h 37 °C exposed (HS) groups revealed: (**A**) Volcano plot of differentially expressed genes (DEGs; red: upregulated, FDR < 0.05, log_2_FC ≥ 1; blue: downregulated, FDR < 0.05, log_2_FC ≤ −1; green: non-significant), with counts of significant DEGs indicated; (**B**) Heatmap depicting global gene expression differences; and (**C**) Bubble plot of the top 20 enriched KEGG pathways (bubble size: number of DEGs; color: −log_10_(FDR)). Enriched pathways encompass protein homeostasis, immune and stress signaling, and metabolic processes.

Differential expression analysis identified 1446 significantly differentially expressed genes (DEGs), of which 639 were upregulated and 807 downregulated (Figure 4A). Hierarchical clustering of these DEGs showed clear grouping by experimental condition and high consistency among biological replicates (Figure 4B), confirming the robustness of the transcriptional response.

KEGG pathway enrichment analysis demonstrated that the DEGs were significantly enriched in multiple biological processes (Figure 4C). These pathways were primarily associated with: (i) protein homeostasis and endoplasmic reticulum stress, as evidenced by enrichment in “Protein processing in endoplasmic reticulum”, suggesting activation of the unfolded protein response; (ii) immune and inflammatory regulation, including the “Toll and Imd signaling pathway” and “PI3K-Akt signaling pathway”, reflecting innate immune activation; (iii) stress signal transduction, particularly the “MAPK signaling pathway”, implicating its role in heat stress signaling; (iv) metabolic reprogramming, with enriched pathways such as “Glycolysis/Gluconeogenesis”, “Pyruvate metabolism”, “Citrate cycle (TCA cycle)”, and “Purine metabolism”, indicating adaptive shifts in energy and biosynthetic metabolism; and (v) cell adhesion and communication, including “ECM–receptor interaction” and “Axon guidance”, which may influence hemocyte migration and intercellular dynamics.

### 3.4. Gene Ontology Enrichment Analysis of DEGs

**Figure 5:**
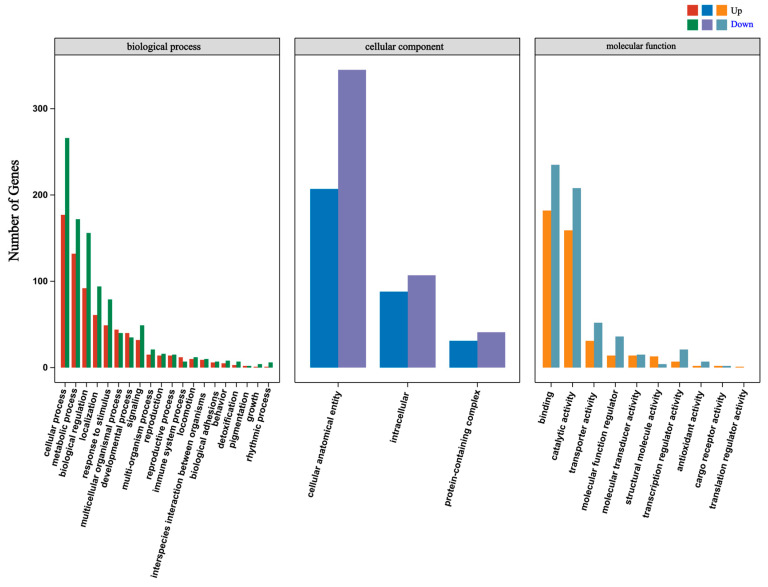
GO functional enrichment analysis of DEGs in *P. clarkii* hemocytes under acute heat stress. The bar chart depicts the distribution of upregulated and downregulated DEGs across second-level terms within the three main Gene Ontology (GO) categories: biological process, cellular component, and molecular function. In the biological process category, upregulated DEGs were predominant in terms related to cellular defense and adaptation, including “response to stimulus,” “immune system process,” “detoxification,” and “signaling.” Enrichment patterns in the cellular component and molecular function categories reflect corresponding transcriptional changes associated with cellular structure and protein activity.

To characterize the functional landscape of DEGs in hemocytes under acute heat stress, GO enrichment analysis was performed. Upregulated DEGs outnumbered downregulated ones across all three GO categories. In the Biological Process category, DEGs were significantly enriched in cellular process, metabolic process, response to stimulus, and immune system process (Figure 5). Further refined enrichment was observed in gluconeogenesis, cellular iron ion homeostasis, iron ion transport, and innate immune response (Figure S2), indicating metabolic reprogramming and activation of iron-related oxidative stress defense. Enrichment was also detected for circadian rhythm-associated terms, including “circadian behavior” and “locomotor rhythm” (Figure S2), suggesting potential disruption of biological rhythms under thermal stress.

In the Cellular Component category, enriched terms were primarily associated with cell anatomical entities, intracellular regions, and protein-containing complexes, pointing to a transcriptional response concentrated on intracellular structures and protein interaction networks. For Molecular Function, significant enrichment was found for binding, catalytic activity, transporter activity, molecular function regulation, and transcription/translation regulatory activities (Figure 5), reflecting a coordinated molecular strategy involving enhanced catalysis, regulated transport, and multi-layered gene expression control. Together, these results indicate that acute heat stress induces systemic functional reprogramming in hemocytes, integrating metabolic, immune, and regulatory pathways.

### 3.5. KEGG Pathway Enrichment Analysis

KEGG enrichment analysis was conducted to identify pathway-level responses to heat stress in hemocytes. Enriched pathways spanned five major functional modules: Cellular Processes, Environmental Information Processing, Genetic Information Processing, Metabolism, and Organismal Systems.

**Figure 6:**
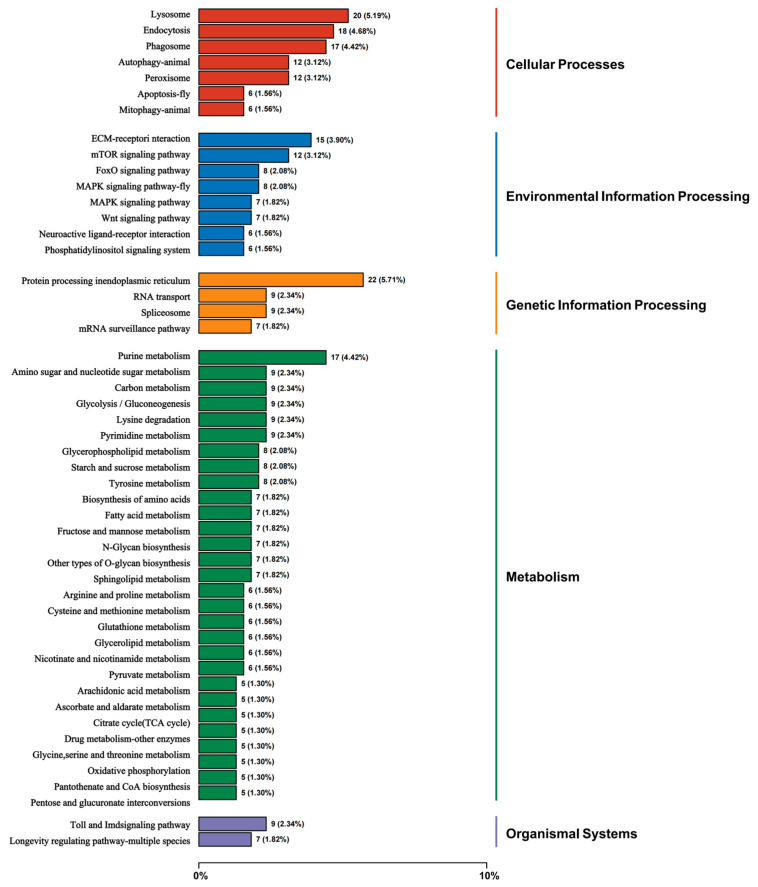
KEGG pathway classification of DEGs in *P. clarkii* hemocytes under acute heat stress. DEGs were systematically assigned to five major functional categories (Cellular Processes, Environmental Information Processing, Genetic Information Processing, Metabolism, Organismal Systems) based on the KEGG database. The bar chart shows the percentage of DEGs annotated to each pathway (relative to all annotated DEGs). Pathways with the highest representation include “Protein processing in endoplasmic reticulum” (5.71%), “Endocytosis” (4.68%), “Phagosome” (4.42%), and “Purine metabolism” (4.42%), indicating extensive transcriptional reprogramming in key processes such as protein homeostasis, cellular internalization, immune clearance, and basal metabolism.

Within the Cellular Processes module, DEGs were enriched in pathways associated with protein homeostasis and intracellular degradation, including “Protein processing in endoplasmic reticulum” (22 DEGs, 5.71%), “Lysosome” (20 DEGs, 5.19%), “Phagosome” (17 DEGs, 4.42%), and “Autophagy—animal” (12 DEGs, 3.12%) (Figure 6). Key signaling pathways were also significantly engaged. Within Environmental Information Processing, the central “MAPK signaling pathway” was markedly enriched, underscoring its role in stress signal transduction. At the Organismal Systems level, enrichment of both the “Toll and Imd signaling pathway” and the “Circadian rhythm—fly” pathway was observed (Figure S3), confirming systemic immune activation and providing evidence for disruption of the core circadian clock. In summary, KEGG pathway analysis demonstrates that acute heat stress triggers comprehensive functional reorganization in hemocytes, characterized by concurrent activation of protein stress and immune clearance pathways, extensive metabolic reprogramming, engagement of conserved stress signaling, and disruption of circadian rhythms.

### 3.6. GSEA Reveals Systemic Functional Imbalance in Hemocytes Under Heat Stress

**Figure 7:**
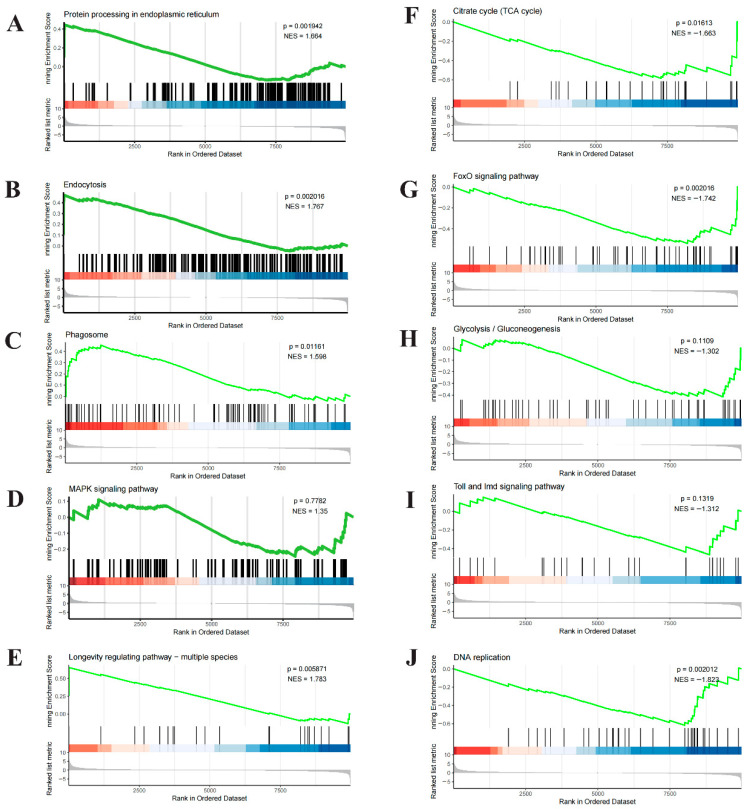
Gene set enrichment analysis (GSEA) of key pathways in *P. clarkii* hemocytes under acute heat stress. GSEA of representative KEGG pathways assessed their coordinated expression changes. Each panel shows the enrichment plot with gene ranking and core statistics (normalized enrichment score, NES; nominal *p*-value). (**A**–**C**) Significantly upregulated pathways (NES > 0, *p* < 0.05), including “Protein processing in endoplasmic reticulum,” “Endocytosis,” and “Phagosome,” indicate activation of protein quality control and immune clearance. (**D**–**J**) Pathways that were downregulated or not significant (NES < 0 or *p* ≥ 0.05), such as “MAPK signaling,” “Citrate cycle (TCA cycle),” “FoxO signaling,” and “Glycolysis/Gluconeogenesis,” suggest suppressed or uncoordinated transcription in basal metabolism, signaling, and proliferation.

To systematically evaluate pathway-level transcriptional responses, Gene Set Enrichment Analysis (GSEA) was performed on hemocyte transcriptomes. This analysis revealed a pronounced functional imbalance, characterized by the concurrent enrichment of defense-related pathways and suppression of core metabolic and biosynthetic processes (Figure 7; Table S3 lists detailed enrichment statistics).

Pathways associated with cellular defense and stress responses were significantly enriched (normalized enrichment score [NES] > 1.5, FDR < 0.05), including “Protein processing in endoplasmic reticulum” (Figure 7A), “Endocytosis” (Figure 7B), “Phagosome” (Figure 7C), and “Longevity regulating pathway” (Figure 7E). In contrast, pathways involved in cell cycle progression and anabolic metabolism were broadly suppressed (NES < −1.5, FDR < 0.05). Notably, “DNA replication”, “FoxO signaling”, “Citrate cycle (TCA cycle)” (Figure 7F), and “Pyruvate metabolism” (Figure 7G) were among the most downregulated pathways. Suppression was also observed in biosynthetic routes such as “Pantothenate and CoA biosynthesis”. The “MAPK signaling pathway” did not exhibit statistically significant coordinated changes (NES = 1.35, FDR = 0.778; Figure 7D).

Collectively, these results indicate that acute heat stress induces a state of functional imbalance in hemocytes, in which enhanced activation of defense and stress-response pathways coincides with a coordinated suppression of energy metabolism and biosynthetic capacity.

### 3.7. Expression Patterns of Key Pathway Genes

**Figure 8:**
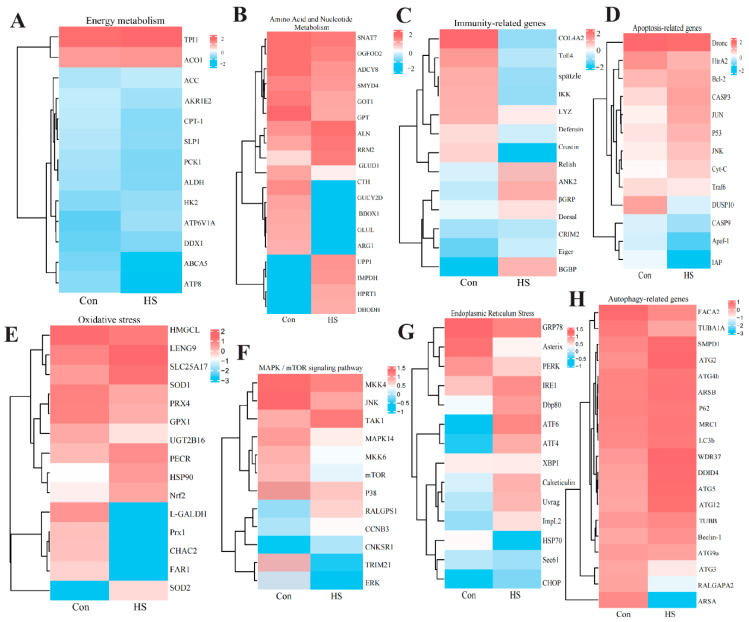
Heatmap analysis of key pathway gene expression in *P. clarkii* hemocytes under acute heat stress. The heatmap depicts the expression patterns of differentially expressed genes across eight representative pathways: (**A**) Energy metabolism, (**B**) Amino acid and nucleotide metabolism, (**C**) Immune, (**D**) Apoptosis, (**E**) Oxidative stress, (**F**) MAPK/mTOR signaling, (**G**) Endoplasmic reticulum stress, and (**H**) Autophagy. Expression values are log_2_-transformed, with red indicating upregulation and blue indicating downregulation relative to the control (Con). Expression profiles for the control (Con) and heat-stressed (HS) groups are displayed alongside.

To complement the pathway-level findings from GSEA, the expression patterns of DEGs across eight core biological processes were examined using hierarchical clustering (Figure 8). Heatmap analysis revealed a coordinated transcriptional reprogramming, characterized by global downregulation of genes involved in basal metabolism and immune effector functions, coupled with selective upregulation of genes mediating cellular stress responses.

Specifically, genes associated with energy metabolism (*HK2*, *ACO1*, *ATP6V1A*) and immune effectors (*Toll4*, *Crustin*, *LYZ*) were predominantly downregulated (Figure 8A,C). In contrast, genes involved in cellular stress and quality control were markedly upregulated. These included autophagy-related genes (*ATG5*, *ATG12*, *LC3b*, *Beclin-1*; Figure 8H), components of the endoplasmic reticulum stress pathway (*PERK*, *GRP78*, *XBP1*, *CHOP*; Figure 8G), and pro-apoptotic factors (*CASP3*, *Cyt-C*, *P53*; Figure 8D). Select antioxidant genes (e.g., *HSP90*, *PRX4*) were also induced (Figure 8E).

More complex regulation was observed in certain pathways. Most components of the MAPK/mTOR signaling network (e.g., *MKK4/6*, *MAPK14/p38*, *mTOR*) were downregulated (Figure 8F), whereas genes involved in amino acid and nucleotide metabolism exhibited mixed expression patterns, with both induced and repressed genes present (Figure 8B).

Collectively, this gene-level analysis supports and extends the pathway-level findings, indicating that acute heat stress reprograms hemocyte transcription toward suppressed basal metabolism and immunity alongside activated protein quality control, autophagy, and apoptosis pathways.

### 3.8. qRT-PCR Validation of Key Differentially Expressed Genes

**Figure 9:**
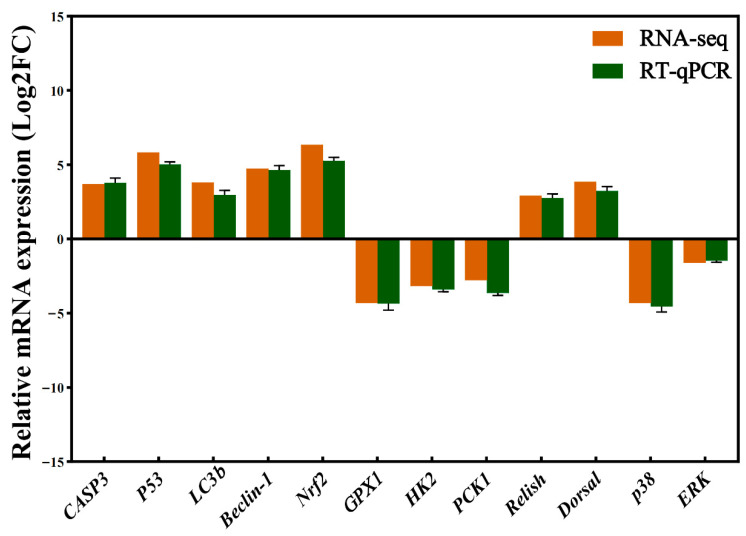
qRT-PCR validation of key gene expression in *P. clarkii* hemocytes under acute heat stress. The mRNA expression of 12 selected key genes was validated by qRT-PCR. The genes involved in apoptosis (*CASP3*, *P53*), autophagy (*LC3b*, *Beclin-1*), oxidative stress (*Nrf2*, *GPX1*), energy metabolism (*HK2*, *PCK1*), and immune signaling (*Relish*, *Dorsal*, *p38*, *ERK*) were quantified after heat exposure. Data are presented as log_2_(fold change) relative to the control group, expressed as mean ± SEM (n = 3).

To independently validate the transcriptome data, qRT-PCR was performed on 12 key DEGs representing major functional pathways. The results showed high concordance with the RNA-Seq expression trends, confirming the reliability of the sequencing data (Figure 9).

The qRT-PCR results validated specific transcriptional alterations across functional categories. Genes involved in cellular stress responses were consistently upregulated, including pro-apoptotic factors (*CASP3*, *P53*) and autophagy markers (*LC3b*, *Beclin-1*). In redox regulation, a potential decoupling was observed: the master regulator *Nrf2* was induced, whereas its downstream target *GPX1* was suppressed. Conversely, genes associated with core energy metabolism (*HK2*, *PCK1*) were downregulated, independently confirming the transcriptional suppression of these pathways.

Divergent regulation was evident in immune-related signaling pathways. Key transcription factors of the innate immune NF-κB pathway (*Relish*, *Dorsal*) were significantly upregulated. In contrast, core components of the MAPK pathway (*p38*, *ERK*) were downregulated, indicating pathway-specific responses to heat stress. In summary, the qRT-PCR validation supports the transcriptomic findings, demonstrating that acute heat stress induces a coordinated transcriptional reprogramming characterized by concurrent activation of apoptosis and autophagy, suppression of antioxidant effector genes and energy metabolism, and divergent regulation of innate immune versus MAPK signaling.

## 4. Discussion

Acute heat stress is a primary driver of mass mortality in crustacean aquaculture [4,20], yet the molecular cascade linking thermal shock to immune collapse remains poorly defined. In this study, we provide multi-level evidence that acute heat stress induces systemic immunosuppression in *P. clarkii* through a coordinated pathogenic sequence: an initial redox collapse triggers targeted apoptosis of granular hemocytes, while concurrent transcriptional reprogramming creates a state of metabolic–immune decoupling that renders surviving immune cells functionally impaired.

### 4.1. Redox Collapse as the Initiating Event

Exposure to 37 °C provoked a severe disruption of redox homeostasis in *P. clarkii*, evidenced by accumulation of ROS and H_2_O_2_ alongside suppression of SOD and CAT activities (Figure 3). This pattern of elevated oxidative products with concomitant antioxidant suppression indicates failure of the redox-buffering network rather than a compensatory response. The coordinated suppression at both enzymatic and transcriptional levels (e.g., *GPX1* downregulation; Figure 8E) suggests that acute thermal stress overwhelms antioxidant capacity before adaptive upregulation can occur. The functional consequences were evident in the significant accumulation of MDA, a lipid peroxidation product known to activate stress-related pathways [46,47,48]. Consistent with this, we observed upregulation of the pro-apoptotic gene *P53* (Figure 8D), supporting involvement of an oxidative stress–p53–apoptosis axis.

Comparative evidence across aquatic species reinforces the centrality of redox collapse in thermal stress responses. In the ark shell Scapharca subcrenata, acute heat stress induced a transient rise followed by a sharp decline in SOD and CAT activities within 12 h, coincident with elevated MDA and increased apoptosis [49]. Notably, chronic exposure in the same species allowed sustained antioxidant activity, underscoring that exposure duration dictates the capacity for redox recovery. A parallel distinction is observed in rainbow trout (*Oncorhynchus mykiss*), where chronic sublethal warming induces compensatory upregulation of hepatic antioxidant genes [4,50]. Together, these cross-species comparisons highlight that an instantaneous failure of redox-buffering is a defining feature of acute thermal shock, as observed in *P. clarkii*.

The link between heat-induced oxidative stress and immune impairment is further supported by evidence from other invertebrate taxa. In the top shell, *Turbo sazae*, elevated temperature simultaneously increases ROS and decreases phagocytic capacity [51]. Similar patterns have been reported in the Pacific oyster, *Crassostrea gigas* [52], and the hard clam, *Meretrix petechialis* [53], where heat stress both weakens immune responses and exacerbates oxidative damage.

Sex-specific differences also merit consideration: in the green crab *Carcinus maenas* exposed to simulated marine heatwaves, females exhibited significantly elevated SOD and glutathione reductase activities compared with males [54], indicating that antioxidant capacity varies with physiological factors. Collectively, these findings support the conclusion that rapid, uncompensated redox collapse serves as a critical initiating event for the downstream immunopathological cascade observed in this study.

### 4.2. Granular Hemocyte Apoptosis as the Cellular Basis of Immune Deficiency

Heat stress reduced total hemocyte count by approximately 25% and specifically decreased granulocytes (Figure 2A,C), identifying this cell type as a primary cellular target. Granulocytes are central to crustacean cellular immunity, functioning as the principal effectors of encapsulation, melanization, and phagocytosis [55,56]. Their selective depletion, therefore, represents a significant erosion of immune capacity.

The mechanistic basis for this selective vulnerability likely resides in intrinsic properties of granulocytes. These cells are enriched in the pro-phenoloxidase system, a critical immune pathway that generates cytotoxic intermediates during pathogen clearance [7,56,57]. Under oxidative stress, dysregulation of this system can lead to self-toxicity, rendering granulocytes particularly susceptible to apoptosis when redox balance is compromised [50,58]. The concurrent upregulation of pro-apoptotic genes (*CASP3*, *P53*) and the ER stress marker *CHOP* (Figure 8D,G) supports the interpretation that synergistic oxidative and ER stress activate the mitochondrial apoptotic pathway within this subpopulation.

Our findings align with observations in other crustacean species. In *Litopenaeus vannamei*, heat stress-induced hemocyte apoptosis has been linked to reduced phagocytic activity and increased susceptibility to *Vibrio infection* [13,23,59,60]. In the spiny lobster *Jasus lalandii*, chronic exposure to elevated temperature combined with hypercapnia resulted in significantly reduced total hemocyte counts following bacterial challenge, indicating that thermal stress compromises the capacity to mount an effective cellular immune response [61]. A previous study in *P. clarkii* exposed to 35 °C reported significantly reduced activities of T-SOD and lysozyme in hemolymph, accompanied by altered disease progression following white spot syndrome virus challenge [62]. Although viral replication was suppressed at elevated temperature, the reduced immune enzyme activity indicated compromised baseline immune competence.

This pattern of stress-induced apoptosis extends beyond crustaceans. Heat stress has been shown to induce apoptosis in fish immune organs [51] and in bivalve gill tissues [52], suggesting that the cellular response to thermal insult is conserved across diverse aquatic taxa. The intensity of the stress applied here, which exceeded a protective threshold, likely contributed to the transition from homeostatic regulation to a deleterious apoptotic program that eliminates essential immune effectors. Thus, the available evidence supports a model in which granulocyte apoptosis represents a central cellular event linking initial redox collapse to systemic immune failure under acute thermal stress.

### 4.3. Transcriptional Reprogramming and Metabolic-Immune Decoupling

Beyond the cellular consequences of apoptosis, RNA-Seq analysis revealed extensive transcriptional reprogramming in hemocytes. A total of 1446 differentially expressed genes exhibited a paradoxical pattern: coordinated upregulation of defense-related pathways (ER stress response, autophagy, phagosome) alongside broad suppression of core metabolic functions (TCA cycle, glycolysis, pyruvate metabolism) (Figure 4, Figure 6 and Figure 7). This pattern suggests a resource-sparing strategy that, while ostensibly adaptive, creates a critical vulnerability.

Activation of ER stress markers (*GRP78*, *XBP1*) and autophagy genes (*ATG5*, *LC3b*) indicates a concerted response to protein misfolding and organelle damage [29,63]. However, simultaneous suppression of energy metabolism pathways implies that the energetic demands of these protective responses may not be met. This metabolic–immune decoupling, which reflects heightened transcriptional alert with constrained biosynthetic capacity, may explain the observed functional paralysis: despite upregulation of immune signaling components, activities of key immune enzymes (ACP, AKP) were significantly reduced (Figure 2E,F). One plausible mechanism is that ATP depletion, resulting from suppressed energy metabolism, restricts the synthesis, modification, and secretion of effector proteins, thereby rendering transcriptional immune activation ineffective.

This phenomenon is not unique to *P. clarkii*. In the intestine of heat-stressed *P. clarkii* at 35 °C, antioxidant and immune-related enzyme activities initially increased but subsequently declined after 72 h, suggesting that sustained thermal stress eventually overwhelms compensatory mechanisms [64]. In *Scapharca subcrenata*, acute heat stress induced a rapid but transient increase in SOD and CAT activities followed by a sharp decline within 12 h, while apoptosis rates continued to rise [49]. In *Artemia franciscana*, *Hsp70* knockdown resulted in differential expression of carbohydrate metabolism genes and altered glycogen and trehalose levels, indicating that heat shock proteins play a role in energy homeostasis [65]. A similar signaling-function dissociation has been observed in chronically heat-stressed Siberian sturgeon (*Acipenser baerii*), where upregulated immune gene transcription occurred alongside increased tissue damage and apoptosis [66]. The downregulation of *HK2* and *PCK1* in our qRT-PCR validation (Figure 9) independently confirms that energy metabolism is transcriptionally suppressed, consistent with a state of metabolic crisis. Collectively, these data support the interpretation that metabolic-immune decoupling represents a key molecular mechanism contributing to the immunosuppressive phenotype.

### 4.4. An Integrated Pathogenic Model

**Figure 10:**
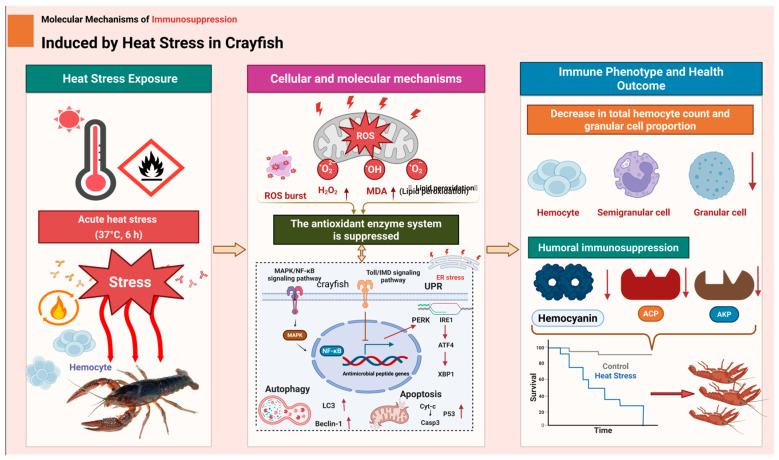
A schematic diagram summarizing the integrated mechanisms of immunosuppression induced by acute heat stress in *P. clarkii*. The diagram integrates multi-omics findings from this study to illustrate the cascade of events following 6 h exposure to 37 °C. The central panel depicts key cellular and molecular mechanisms, including oxidative burst, suppression of antioxidant enzymes, hemocyte depletion, endoplasmic reticulum stress, dysregulation of MAPK/NF-κB and Toll/IMD signaling, activation of autophagy and apoptosis, and alterations in humoral immune components (e.g., hemocyanin, ACP, AKP). These alterations collectively drive the immunosuppressive phenotype (right panel), characterized by humoral immunosuppression and increased mortality, as outlined from exposure (left panel) to outcome.

Based on the multi-level evidence presented, we propose an integrated model (Figure 10) in which acute heat stress drives systemic immunosuppression through a cascade of interconnected events. The initial thermal insult induces a rapid redox crisis characterized by ROS accumulation and antioxidant suppression. This oxidative insult, together with ER stress resulting from protein misfolding, triggers selective apoptosis of granulocytes, depleting a critical effector cell population and eroding the structural basis of cellular immunity [59,67]. Concomitantly, a global transcriptional reprogramming activates stress and immune pathways, but this response is undermined by systemic suppression of core energy metabolism, creating a state of metabolic-immune decoupling that renders surviving immune cells functionally impaired. The synergy between targeted immune cell loss and functional paralysis of the remaining immune apparatus establishes a self-reinforcing pathological cycle, culminating in host defense collapse.

This model aligns with emerging frameworks for understanding environmental stress-induced immunopathology. In bivalves, combined oxidative and ER stress have been implicated in apoptosis and immunosuppression following exposure to harmful algal toxins [68], suggesting that convergent stress pathways may produce similar outcomes across distinct taxonomic groups and stressor types.

### 4.5. Implications and Future Perspectives

Building on the integrated model proposed above, this study establishes a coherent mechanistic framework linking acute heat stress to systemic immunocollapse in *P. clarkii*. The model highlights three critical events: redox imbalance, granulocyte apoptosis, and metabolic-immune decoupling. It advances our understanding of heat-induced mortality in farmed crustaceans and offers a conceptual framework for how environmental stressors compromise aquatic host defense.

The findings provide clear implications for aquaculture. The identification of granulocytes as the primary cellular target of heat stress offers a precise phenotypic marker for selective breeding. Strains with enhanced antioxidant capacity or reduced apoptotic sensitivity could be developed to improve stock resilience. Additionally, these results support the development of functional feeds supplemented with targeted antioxidants or ER stress mitigators, representing a practical nutritional strategy to alleviate heat-induced immunotoxicity. However, this study focused on acute thermal shock; the long-term acclimation response to sublethal warming, therefore, remains an open question.

Several important research directions emerge. Future studies should integrate transcriptomic and epigenetic analyses over extended periods to distinguish adaptive from maladaptive pathways under chronic warming. While omics analyses identified key regulatory genes such as *P53* and *XBP1*, their causal roles require validation through in vivo gene editing or RNA interference. Single-cell sequencing of hemocyte subpopulations would further clarify cell-type-specific vulnerability. Finally, to reflect realistic aquaculture conditions, multi-stressor experiments combining heat with hypoxia or pathogens are needed to evaluate compound effects on immune competence. In summary, this work delineates the immunopathological sequence triggered by heat stress in crayfish, bridges molecular mechanisms to organismal vulnerability, and provides a scientific foundation for developing integrated strategies to enhance thermal resilience in aquaculture under climate change.

## 5. Conclusions

This study elucidates how acute heat stress drives systemic immune collapse in *P. clarkii*. The initial thermal shock induces a dual redox crisis, which consists of an oxidative burst coupled with suppressed antioxidant defenses, leading to lipid peroxidation and endoplasmic reticulum stress. These damages subsequently converge to trigger targeted apoptosis of granulocytes, thereby depleting this pivotal immune cell population. Transcriptomic profiling reveals a maladaptive reprogramming characterized by upregulation of stress and defense pathways alongside downregulation of core energy metabolism, resulting in a critical decoupling of immune signaling from effector capacity. Our integrated analysis demonstrates that the synergy between metabolic paralysis and specific immune cell loss is central to host collapse. Collectively, these findings establish a mechanistic and targetable framework for enhancing thermal resilience, providing direct support for developing apoptosis-resistant breeding lines and functional feeds to mitigate heat-induced losses in crayfish aquaculture.

## Figures and Tables

**Table 1:** Primer sequences used for quantitative real-time PCR (qRT-PCR).

Gene Name	Primer Sequence (5′ → 3′)	Product Length (bp)
18S	F: ACCGATTGAATGATTTAGTGAG	131
	R: TACGGAAACCTTGTTACGAC	
CASP3	F: CCCGACTTAAATGGAATAG	111
	R: TCGCACGTAGAAAAGAGAT	
P53	F: GCCGTTTCTTATTCTCCAT	149
	R: AATCTTAGCATCAGCCCAC	
LC3b	F: GGAACAACATCCAAATAAA	227
	R: CATAAACCTGAGCAAGAGT	
Beclin-1	F: GCGTGAGAAATGGAAACTT	219
	R: TGGTGGCATCGATAACTAG	
Nrf2	F: CAGGAGGAAGGTCATTGTT	171
	R: CAAGTAATTGGGAGGTTCA	
GPX1	F: ACACTGCCTGACCTGCCTT	267
	R: TTCCCCTTCTGGTTCTCCT	
HK2	F: CGTTTCCACCCTCATTTCC	113
	R: GCAGCACCACGTCCACTAC	
PCK1	F: ATCTCTGCCATTCTGTTTG	141
	R: TACCTTGCCCTTGTATTCT	
Relish	F: GGATGTGGCATTCAGGGTA	257
	R: GGTGTAGGGCAGTTTCGCT	
Dorsal	F: GACGGGATGATAGAATGGG	191
	R: AGAGGCAGGTACTGGAACG	
P38	F: GTAATGATAGTGAGGTAGC	165
	R: GAGGTTGGTGAGTAGAAGA	
ERK	F: CACAGAACGAACAAATAAG	161
	R: GTACAATGAAAGGGACATC	

## Data Availability

The raw transcriptome data have been uploaded to the NCBI database under the accession number PRJNA1381293.

## Supplementary Materials

The following supporting information can be downloaded at: https://www.mdpi.com/article/10.3390/biology15070582/s1. Figure S1: Principal component analysis (PCA) of hemocyte transcriptomes from control and heat-stressed *P. clarkii*. PCA was performed on the gene expression profiles of all samples (n = 6) based on RNA-Seq data to visualize global transcriptional differences. Each point represents an individual sample, labeled as Con: control; HS: heat-stressed. PC1 and PC2 explain 43.64% and 17.25% of the variance, respectively. The clear separation between the control and heat-stressed groups in the PCA space indicates a systemic alteration of the hemocyte transcriptome induced by heat stress. Figure S2: Heatmap of selected differentially expressed genes and their enriched GO terms in hemocytes under heat stress. The heatmap displays expression changes of a subset of representative differentially expressed genes between heat-stressed and control groups. Color intensity corresponds to log_2_ fold change (log_2_FC), with red indicating upregulation and blue indicating downregulation. GO terms significantly enriched among these genes are listed on the right, encompassing biological processes such as gluconeogenesis, cellular iron ion homeostasis, innate immune response, and rhythmic behavior. This pattern suggests coordinated transcriptional regulation across metabolism, ion balance, and immune-circadian functions in response to heat stress. Figure S3: Expression patterns of differentially expressed genes across selected KEGG pathways in hemocytes under heat stress. The heatmap displays differentially expressed genes involved in five representative KEGG pathways: Glycolysis/Gluconeogenesis, Cytosolic DNA-sensing pathway, MAPK signaling pathway, Purine metabolism, and Circadian rhythm – fly. Rows represent individual genes (denoted by LOC identifiers), and columns represent pathway categories. Color intensity reflects standardized gene expression levels, with red and blue indicating upregulation and downregulation, respectively. The presence of some genes across multiple pathways suggests their potential multifunctional regulatory roles. This visualization highlights coordinated expression changes across distinct functional modules. Table S1: Summary of transcriptomic sequencing data quality for *P. clarkii*. Table S2: Summary of sequencing quality metrics for *P. clarkii* under different treatments. Table S3: Significantly enriched KEGG pathways identified by GSEA in the hemocyte of *P. clarkii* under heat stress.
