# Supplementary material for: Transcriptome Analysis Provides New Insight into Apoptosis and Immunosuppression in Procambarus clarkii After Exposure to High Temperature
Source: Biology (Basel). 2026 Apr 5;15(7):582. doi: 10.3390/biology15070582 (PMC13072406; doi:10.3390/biology15070582)
Supplement: Supplementary file 1 [file biology-15-00582-s001.zip › biology-4155036-supplementary.pdf]

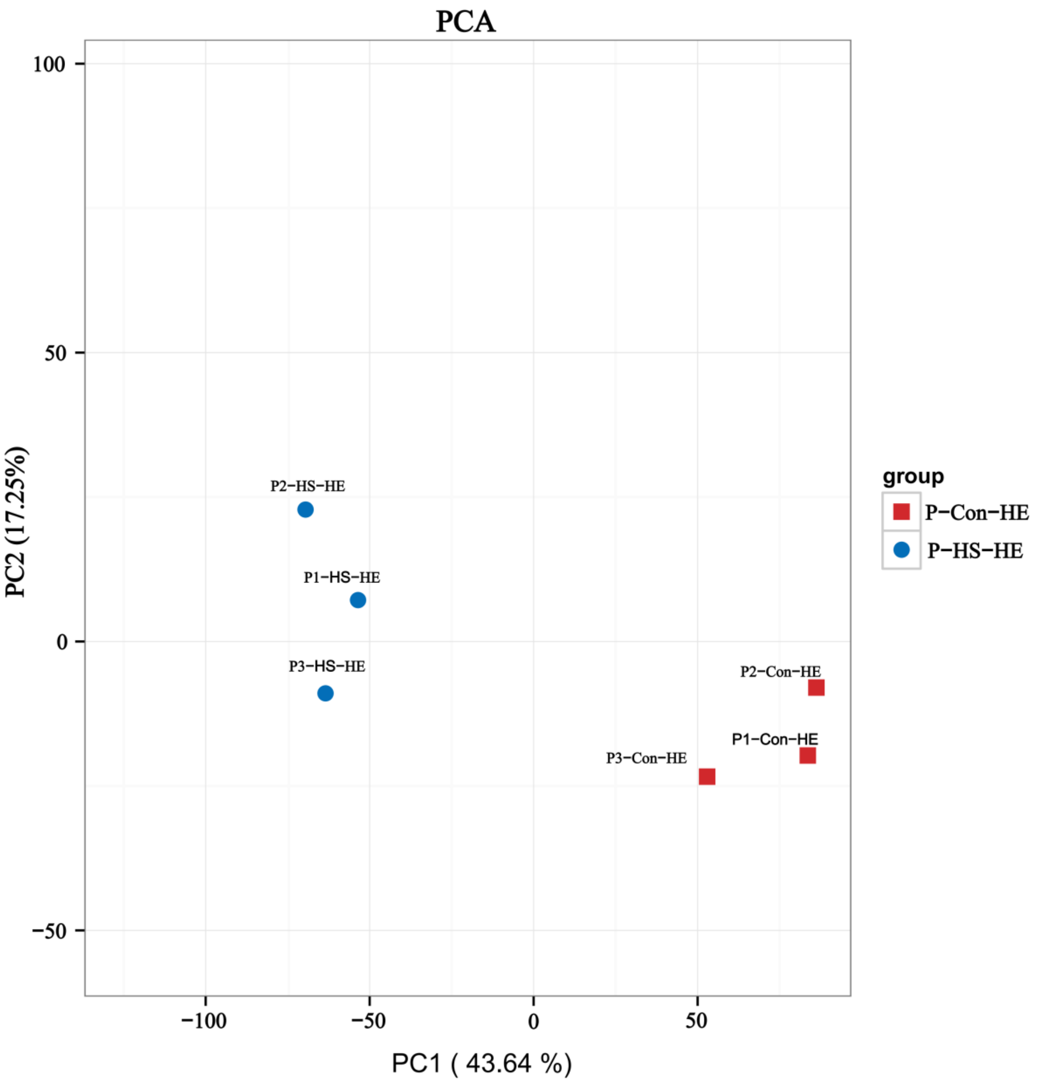


Figure S1. Principal component analysis (PCA) of hemocyte transcriptomes from control and heat-stressed *P. clarkii*.

PCA was performed on the gene expression profiles of all samples (n = 6) based on RNA-Seq data to visualize global transcriptional differences. Each point represents an individual sample, labeled as Con: control; HS: heat-stressed. PC1 and PC2 explain 43.64% and 17.25% of the variance, respectively. The clear separation between the control and heat-stressed groups in the PCA space indicates a systemic alteration of the hemocyte transcriptome induced by heat stress.

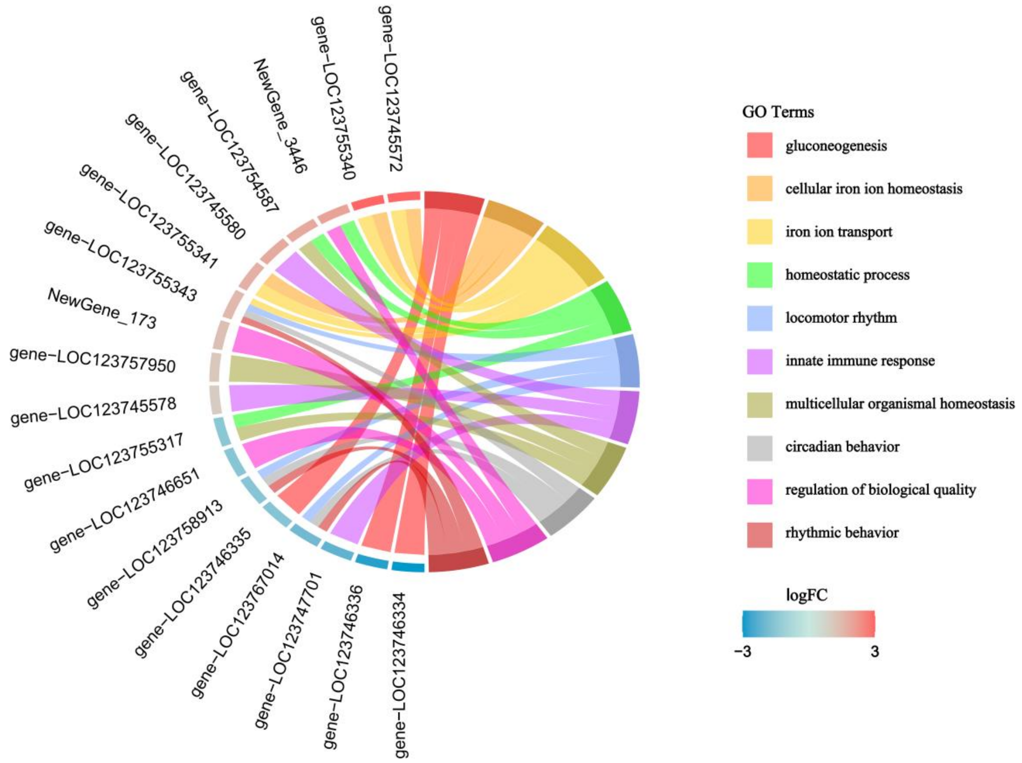


Figure S2. Heatmap of selected differentially expressed genes and their enriched GO terms in hemocytes under heat stress.

The heatmap displays expression changes of a subset of representative differentially expressed genes between heat-stressed and control groups. Color intensity corresponds to $\log_2$ fold change ($\log_2$FC), with red indicating upregulation and blue indicating downregulation. GO terms significantly enriched among these genes are listed on the right, encompassing biological processes such as gluconeogenesis, cellular iron ion homeostasis, innate immune response, and rhythmic behavior. This pattern suggests coordinated transcriptional regulation across metabolism, ion balance, and immune-circadian functions in response to heat stress.

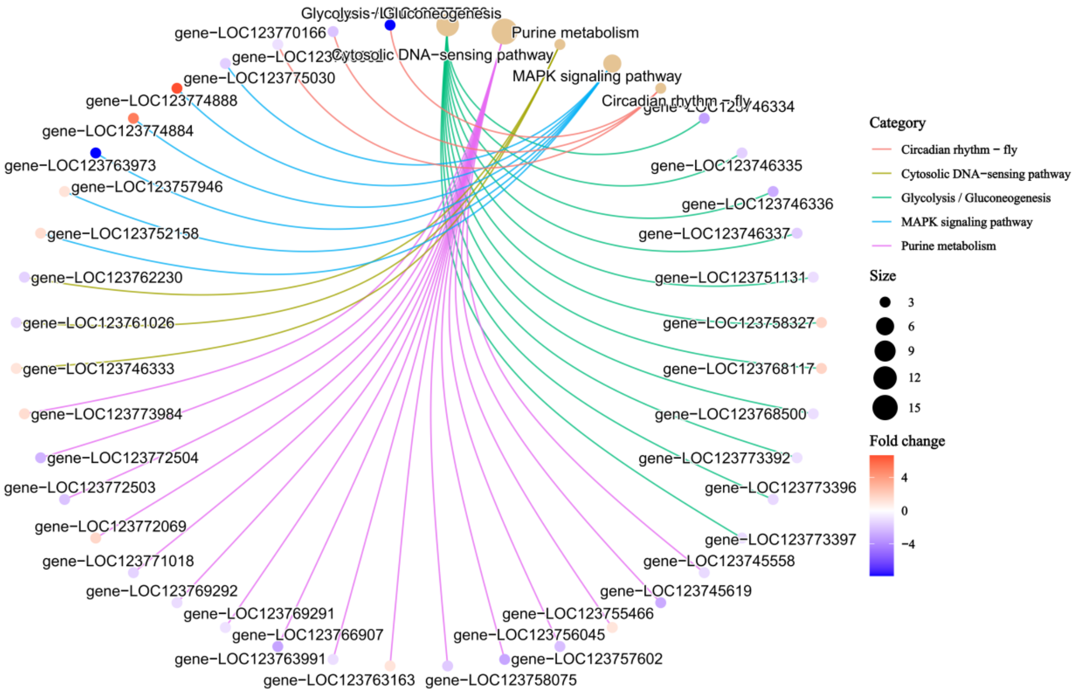


Figure S3. Expression patterns of differentially expressed genes across selected KEGG pathways in hemocytes under heat stress.

The heatmap displays differentially expressed genes involved in five representative KEGG pathways: Glycolysis/Gluconeogenesis, Cytosolic DNA-sensing pathway, MAPK signaling pathway, Purine metabolism, and Circadian rhythm – fly. Rows represent individual genes (denoted by LOC identifiers), and columns represent pathway categories. Color intensity reflects standardized gene expression levels, with red and blue indicating upregulation and downregulation, respectively. The presence of some genes across multiple pathways suggests their potential multifunctional regulatory roles. This visualization highlights coordinated expression changes across distinct functional modules.

**Table S1: Summary of transcriptomic sequencing data quality for *P. clarkii*.**

| Sample ID | Clean Reads | Clean Bases (Gb) | GC Content (%) | Q30 (%) |
|---|---|---|---|---|
| P1-Con-HE | 21,207,712 | 6.34 | 45.02 | 95.09 |
| P2-Con-HE | 21,961,803 | 6.57 | 44.82 | 94.47 |
| P3-Con-HE | 22,669,923 | 6.78 | 45.03 | 94.74 |
| P1-HS-HE | 19,818,830 | 5.93 | 44.05 | 94.28 |
| P2-HS-HE | 22,234,038 | 6.65 | 44.40 | 94.94 |
| P3-HS-HE | 22,801,873 | 6.82 | 43.79 | 94.36 |

**Note:** Sample ID denotes: Con, control group; HS, heat stress group; HE, Hemocyte; P1-P3, biological replicates 1-3. Clean bases are presented in gigabases (Gb). Q30 indicates the percentage of bases with a Phred quality score ≥ 30.

**Table S2: Summary of sequencing quality metrics for *P. clarkii* under different treatments.**

| Sample ID | Clean Reads (Million) | Clean Bases (Gb) | GC (%) | Q20 (%) | Q30 (%) |
|---|---|---|---|---|---|
| P1-Con-HE | 21.21 | 6.34 | 45.02 | 98.29 | 95.09 |
| P2-Con-HE | 21.96 | 6.57 | 44.82 | 98.00 | 94.47 |
| P3-Con-HE | 22.67 | 6.78 | 45.03 | 98.12 | 94.74 |
| P1-HS-HE | 19.82 | 5.93 | 44.05 | 97.93 | 94.28 |
| P2-HS-HE | 22.23 | 6.65 | 44.4 | 98.19 | 94.94 |
| P3-HS-HE | 22.80 | 6.82 | 43.79 | 97.94 | 94.36 |

**Note:** Sample ID: Con, control group; HS, heat stress group; HE, Hemocyte; P1-P3, biological replicates. Clean bases are presented in gigabases (Gb). Q20 and Q30 denote the percentage of bases with Phred quality scores ≥ 20 and ≥ 30, respectively.

**Table S3: Significantly enriched KEGG pathways identified by GSEA in the hemocyte of *P. clarkii* under heat stress.**

**A. Upregulated pathways**

| Pathway ID | Pathway Name | Gene Number | ES (abs) | NES | p-value |
|---|---|---|---|---|---|
| ko03040 | Spliceosome | 134 | 0.585 | 2.175 | 0.00196 |
| ko04141 | Protein processing in endoplasmic reticulum | 136 | 0.445 | 1.664 | 0.00194 |
| ko04213 | Longevity regulating pathway - multiple species | 25 | 0.653 | 1.783 | 0.00587 |
| ko04144 | Endocytosis | 160 | 0.471 | 1.767 | 0.00202 |
| ko04145 | Phagosome | 89 | 0.456 | 1.598 | 0.0116 |
| ko00310 | Lysine degradation | 62 | 0.508 | 1.653 | 0.0118 |
| ko00511 | Other glycan degradation | 20 | 0.577 | 1.524 | 0.0454 |

**B. Downregulated pathways**

| Pathway ID | Pathway Name | Gene Number | ES (abs) | NES | p-value |
|---|---|---|---|---|---|
| ko03030 | DNA replication | 37 | 0.618 | -1.823 | 0.00201 |
| ko04068 | FoxO signaling pathway | 56 | 0.541 | -1.742 | 0.00202 |
| ko00240 | Pyrimidine metabolism | 39 | 0.521 | -1.544 | 0.0142 |
| ko00020 | Citrate cycle (TCA cycle) | 30 | 0.585 | -1.663 | 0.0161 |
| ko00230 | Purine metabolism | 79 | 0.437 | -1.518 | 0.0160 |
| ko00620 | Pyruvate metabolism | 33 | 0.556 | -1.608 | 0.0162 |

| Pathway ID | Pathway Name | Gene Number | ES (abs) | NES | p-value |
|---|---|---|---|---|---|
| ko00770 | Pantothenate and CoA biosynthesis | 16 | 0.653 | -1.636 | 0.0216 |
| ko00760 | Nicotinate and nicotinamide metabolism | 29 | 0.542 | -1.539 | 0.0314 |
| ko04150 | mTOR signaling pathway | 87 | 0.392 | -1.378 | 0.0468 |

Note: Gene set enrichment analysis (GSEA) was performed on transcriptomic data from heat-stressed versus control crayfish hemocyte. The table lists KEGG pathways with a nominal p-value < 0.05, categorized by the direction of enrichment: a positive normalized enrichment score (NES) indicates upregulation under heat stress, while a negative NES indicates downregulation. Columns represent: Pathway ID/Name (KEGG identifier and full name), Gene Number (genes in the set), ES (abs) (absolute enrichment score magnitude), NES (normalized enrichment score), and p-value.
